# Gastric Ulcer and Its Treatment

**Published:** 1894-07-07

**Authors:** A. B. Giles


### GASTRIC ULCER AND ITS TREATMENT.

By A. B. Giles, M.B.

Under the clinical title of gastric ulcer it is usual to describe a group of symptoms usually depending on the lesion known to pathologists as simple, round or perforating ulcer of the stomach, although other varieties of ulcer in this situation are not unknown, and in some cases it is very doubtful whether any actual ulcer exists.

The disease is a common one, especially in the female sex, and between the ages of 20 and 30 years, while in men its incidence is later. Its frequency in domestic servants and young women employed in similar occupations has been ascribed to the abuse of tea, especially when consumed at a high temperature. The peculiar circular terraced appearance so characteristic of this ulcer probably depends, as suggested by Virchow, on the shape of area of blood supply, the plugging of whose vessel is the cause of the lesion; the slough being afterwards digested by the gastric juice. The ulcer is generally situated on the lesser curvature or posterior wall near the pyloric extremity of the stomach, and is usually single.

The chief symptoms produced are pain and vomiting, and as the characters of these are important in diagnosis it will be well to treat them separately.

*Pain* of a gnawing or boring character excited within a few minutes, in most cases by the ingestion of food. It is chiefly felt in the epigastric region, frequently being localised over the ensiform cartilage, where there is often tenderness on pressure. It also radiates to the back, being referred to the area between the scapulæ.

*Vomiting* in cases of gastric ulcer usually follows rapidly on the ingestion of food, and generally gives relief from the pain excited by that act. It is sudden, and usually without much retching, and though the vomited matter may contain nothing abnormal, it is usual for cases of gastric ulcer to suffer to a greater or less extent from hæmatemesis. The character of the blood vomited varies with the conditions in which it takes place. Thus in some cases the vomited matters present a mixture of slightly altered food and small blood clots, which is very characteristic. Again, when the hæmorrhage is slow, and the blood is partly digested before it is rejected by the stomach, it becomes brown and discoloured—the well-known coffee-ground vomit.

When more rapidly shed, as when a large vessel is eroded, the blood retains its natural colour, and, while it may be partly clotted, it is distinguished by its large amount, which, indeed, is a general characteristic of the hæmatemesis of gastric ulcer. Along with hæmatemis, and in rare cases where no vomiting takes place, the stools assume a dark, almost black colour from the presence of altered blood pigments.

Although attended by alarming symptoms, gastric ulcer is fortunately not a very fatal disease, and under careful treatment the patient usually recovers with some failure of digestive power. The chief dangers which may arise may be grouped under the heads of excessive hæmorrhage, perforation, and obstruction of the pylorus.

1. *Hæmorrhage* from the erosion of a large vessel may be so severe as to cause death. It may either come from a vessel in the wall of the stomach, or from some other viscus to which the stomach has become adherent.

2. *Perforation* of the wall of the stomach is also liable to occur, the base of the ulcer often consisting only of the peritoneal coat. It may therefore be excited by hard or indigestible food. The tendency to perforation is lessened by a plastic peritonitis which takes place over the site of the ulcer, causing thickening of the peritoneum and adhesion to other organs of the abdominal walls.

After adhesions have taken place the ulceration may still progress, causing a sinus into a neighbouring viscus, which may end in a severe hæmorrhage, or leading to the formation of a fistula, either with the skin surface or some portion of the intestinal canal. If these results do not follow and perforation takes place into the peritoneal cavity, septic peritonitis from the escape of the stomach contents takes place, and when untreated is usually fatal.

3. *Cicatricial contraction* may, in the process of healing, seriously obstruct the lumen of the alimentary canal, especially when near the pylorus, producing the conditions of fibrous stricture, or occasionally "hour-glass contraction of the stomach" when the stomach is divided into two chambers by a ring of cicatricial tissue. This mechanically interferes with the functions of the organ.

*The treatment of gastric ulcer* divides itself naturally into drugs and diet, and a right understanding of the latter is at least of equal importance to the former if the case is to be brought to a successful termination. Severe hæmatemesis may be treated by the hypodermic injection of ergot, or the administration of acetate of lead and acetic acid. Ice may be given to suck, and in cases where perforation is not feared small fragments may be given to be swallowed. More useful, in so far that it relieves pain and vomiting and gives rest to the stomach while acting as a valuable astringent, is a pill containing half a grain of fresh, solid opium. Great caution must be employed in the administration of stimulants in cases of hæmorrhage, and they should be dispensed with in all but the most critical cases, and then only after arrest is secured.

Of agents given to arrest vomiting, hydrocyanic acid and bismuth in powder or mixture are the chief. The latter is said to owe part of its action to mechanically protecting the floor of the ulcer, and it is evident, if this is the case, the ordinary small doses are insufficient. The sub-nitrate of bismuth may be given in doses of a teaspoonful or more of the powder with perfect safety if it is free from impurities. Purgatives are contra-indicated, but a dose of Carlsbad salts or other alkali in a pint of tepid water taken in the morning in sips obviates the difficulty and also produces a favourable effect on the gastric symptoms. With regard to dietetics it is necessary in the first instance to secure the greatest amount of physiological rest for the affected organs, and in most cases and all severe ones this is best attained by feeding the patient per rectum, at least for a few days. Nutrient enemata are much to be preferred to suppositories, and may with advantage be predigested. Many physicians are in the habit of giving a small quantity, say, a tablespoonful of milk and lime water by the mouth at the same time as the injection, to neutralise the reflex secretion of gastric juice which then takes place and sometimes causes pain.

After a few days' rectal alimentation the patient may receive fluid food by the mouth, such as milk and lime water, and beef-tea in small quantities and frequent intervals, the enemata being continued if these cannot be given in sufficient quantity. From this we may progress in succession to peptonized gruel, infants' foods, boiled bread and milk, fish, chicken, scraped beef, &c., gradually increasing the amount and lengthening the intervals between the meals, being guided by the absence of pain and vomiting. It should be regarded as an axiom that if anything disagrees the patient must be reduced to the next lower round of what has been well termed the "dietary ladder," and the offending substance begun after an interval and in smaller quantity. The diet is thus raised as quickly as is consistent with the patient's comfort to one which is both plentiful and nutritious, for it must not be forgotten that for the repair of the ulcer good food is necessary, though its site requires it to be carefully administered. A too-prolonged course of low diet shows neglect of the first principle, over-attention to the second, and explains the long duration of some otherwise simple cases.

With regard to perforation into the peritoneum, the treatment becomes that of peritonitis, but one can no longer doubt that, if seen sufficiently early, the proper course is to open the abdomen and endeavour to purify the peritoneal cavity, and close the rent in the stomach wall in the same way as for perforation, the result of external injury.*

Cicatricial contraction of the pylorus, if severe, might be dealt with by Loreta's operation, when the fibrous band is stretched by the finger, but in less aggravated cases attention may be necessary to the subsequent dilatation of the stomach.

* Brit. Med. Journ., vol. i., p. 893, p. 1,258.

